# Quantitative comparison of anticancer drug dispersal before and after introducing appropriate preparation procedures during anticancer drug preparation

**DOI:** 10.1186/s40780-022-00250-1

**Published:** 2022-06-15

**Authors:** Yuki Yanagisawa, Kazuo Isama, Tomohiro Kurosu, Yoshiaki Natsume, Toshikazu Seino, Tetsuji Nishimura, Atsushi Yamashita

**Affiliations:** 1 Department of Pharmacy, Yokohama Rosai Hospital, 3211 Kozukue-cho, Kohoku-ku, Yokohama, Kanagawa 222-0036 Japan; 2 Graduate School of Pharmaceutical Sciences, Teikyo Heisei University, 4-21-2 Nakano, Nakano-ku, Tokyo, 164-8530 Japan; 3 Department of Pharmacy, Niigata Prefectural Tsubame Rosai Hospital, 633 Sado, Tubame-shi, Niigata, 959-1228 Japan; 4 Faculty of Pharmaceutical Sciences, Teikyo Heisei University, 4-21-2 Nakano, Nakano-ku, Tokyo, 164-8530 Japan

**Keywords:** Anticancer drug exposure, Preparation procedures, Wipe test, Japanese hospital, Pharmacists, Fluorouracil, Gemcitabine

## Abstract

**Background:**

In Japan, engineering controls for preparing injectable anticancer drugs are inadequate and compliance with appropriate preparation procedures is vital. In this study, we evaluated the effects of adherence to appropriate anticancer drug formulation and packaging procedures on reducing anticancer drug dispersal in clinical practice, especially in Japan.

**Methods:**

We quantitatively evaluated the effectiveness of implementing procedures that were experimentally verified to help reduce the amount of anticancer drug dispersed during preparation based on procedures described in the “Anticancer Drug Preparation Manual.” The target facilities were four regional hub hospitals in the Kanto area. Contamination of sheets and gloves with 5-fluorouracil (5-FU) and gemcitabine (GEM) in a safety cabinet during formulation was evaluated using wipe tests. Subsequently, the proper preparation procedure was shown on a video, training was provided, and the wipe tests were repeated.

**Results:**

Forty-one and 39 pharmacists were engaged in drug preparation before and after intervention, respectively. 5-FU had the highest dispersal per prepared vial on the sheet before intervention. The dispersal amount per prepared vial decreased significantly (*P* = 0.01) after intervention. The amount of GEM dispersed before and after intervention did not differ significantly. However, the percentage of sheets below the detection limit after intervention was 62%, increasing from 46% before intervention. The amount dispersed on gloves was not significantly reduced by proper preparation technique. Although not explicitly noticeable and quantifiable, pharmacists must consider that a significant amount of anticancer drug is dispersed on gloves despite following appropriate preparation procedures.

**Conclusions:**

Quantitative amounts of anticancer drugs dispersed in the preparations of 5-FU and GEM were found in our study. The difference in the amount of contamination before and after intervention was significantly reduced only for the contamination of sheets with 5-FU. There was no decrease in the amount of glove contamination. There was also no difference between medical facilities. Despite following appropriate preparation procedures, dispersed amounts cannot be maintained below the detection limit, indicating the need for a combination of education and engineering controls.

## Background

Healthcare workers responsible for handling anticancer drugs are at risk of occupational exposure. This exposure remains a serious concern, which in many cases leads to health problems due to carcinogenesis or genotoxicity, for example. However, since it is challenging for these healthcare workers to avoid occupational exposure to anticancer drugs, it is essential to minimize such contamination to the maximum possible extent. To prevent exposure to hazardous drugs, such as anticancer agents, the International Standards for Space Omics Processing (ISOPP) Standard and Practice 2007 recommends control measures that implement the hierarchy of hazard control [1]. Hierarchy control is a risk management concept that helps eliminate or minimize exposure to occupational hazards. In a prioritized order, it suggests using machines and equipment effective in preventing exposure, implementing appropriate work practices by workers per the guidelines and procedures established within the organization, and using appropriate personal protective equipment to undertake preventive measures. Combining these approaches, rather than resorting to other preventive measures, can help prevent exposure to anticancer agents.

The National Institute for Occupational Safety and Health (NIOSH), American Society of Health-System Pharmacists (ASHP), and the Exposure Control Guidelines 2019 recommend the use of a closed system drug-transfer device (CSTD) for the preparation of anticancer drugs [2–4]. Since the implementation of CSTDs, reports of high exposure to anticancer drugs and contamination from a wide range of sources have persisted, even though many such drugs are prepared by pharmacists in biological safety cabinets (BSCs) [5]. Furthermore, it has been reported that the use of closed connection devices to prepare and administer anticancer drugs is not prevalent in Japan [6–8]. Therefore, it is vital to use BSCs and personal protective equipment (PPE), appropriately follow the preparation procedures, and provide adequate education to healthcare workers who handle the drugs to achieve the best outcomes. In a previous report, environmental monitoring of cyclophosphamide in six pharmacists engaged in the preparation of anticancer drugs at a single institution showed that environmental exposure in the preparation room was reduced by undertaking several measures, including changes in cleaning methods, the introduction of dispersal equipment, and education on exposure knowledge and techniques [9]. Environmental monitoring, including wipe tests, which are becoming mainstream, is reportedly useful in many studies to investigate the status of environmental exposure [10]. However, it is difficult to measure the amount of anticancer drugs dispersed in many samples and report on the actual situation because of the limited number of target drugs and the high cost of analysis by an external laboratory.

In this study, we focused on implementing appropriate preparation procedures in a multicenter setting and evaluated their usefulness in reducing the amount of anticancer drug dispersal. In addition, 5-fluorouracil (5-FU) and gemcitabine (GEM), which have been used frequently but not in CSTDs, were selected as the investigated drugs. We established an analytical method that enabled us to measure the dispersal of the target anticancer drugs on many collected samples.

Thus, it is extremely important to ensure that administrative controls, including work practices, are prioritized over other measures. However, while many quantitative evaluations have been conducted to highlight the usefulness of CSTDs, few reports have described quantitative evaluation of the usefulness of other suitable preparation procedures, classified under the category of tissue management controls, under actual practice in a multicenter environment. Although Yoshida et al. quantitatively evaluated the effects of handling procedure regulations, and education, among others, on exposure in multiple medical institutions [11], we focused more on the preparation procedure and conducted a quantitative evaluation during the preparation by each pharmacist. Our previous studies found that ideal preparation procedures were not consistently implemented despite each facility having instruction manuals, with no continuous training provided to the concerned healthcare workers on these preparation procedures [3]. Based on the results of a fact-finding survey, we used simulated anticancer drugs to quantitatively evaluate the preparation procedures recommended in the Anticancer Drug Preparation Manual” [10], which describes the conventional procedures for drug preparation in Japan. The following procedures were identified as drawbacks that affect the quantity of drug dispersed: dissolution of multiple vials, recapping, injection of drug solution, and proper air release after preparation [12].

In this study, we aimed to evaluate the outcome of implementing procedures considered practical to reduce the amount of dispersed anticancer drugs, and we compared this reduction during actual operation at multiple facilities.

## Methods and Materials

### Reagents and chemicals

Acetonitrile (ACN, for liquid chromatography–mass spectrometry (LC–MS)), ultrapure water (for LC–MS), and formic acid (98.0%) were purchased from FUJIFILM Wako Pure Chemical Co., Tokyo, Japan. 5-FU (> 99.0%, Tokyo Chemical Industry Co., Ltd., Tokyo, Japan) and GEM (> 98.0%, Tokyo Chemical Industry Co., Ltd.) were used as standards. 5-Chlorouracil (5-CU, > 95.0%, FUJIFILM Wako Pure Chemical Co., Ltd.) was used as the internal standard (IS). Table 1 shows the targeted anticancer drugs and gloves used in the surveyed facilities.

**Table 1:** 5-FU, GEM, and gloves useed in the surveyed facilities

Drug	Facility	Manufacturer	Standard	Appearance	Volume
5-FU	A	Towa Pharmaceutical Co, Ltd, Tokyo, Japan	1000 mg	liquid	20 mL
	B	Towa Pharmaceutical Co, Ltd, Tokyo, Japan	1000 mg/ 250 mg	liquid	20 mL/ 5 mL
	C	Towa Pharmaceutical Co, Ltd, Tokyo, Japan	1000 mg	liquid	20 mL
	D	Towa Pharmaceutical Co, Ltd, Tokyo, Japan	1000 mg	liquid	20 mL
GEM	A	Yakult Honsha Co, Ltd, Tokyo, Japan	1000 mg/ 200 mg	lyophilizer	―
	B	Yakult Honsha Co, Ltd, Tokyo, Japan	1000 mg/ 200 mg	lyophilizer	―
	C	Sandoz K.K., Tokyo, Japan	1000 mg/ 200 mg	liquid	25 mL/ 5 mL
	D	Nichi-Iko Pharmaceutical Co., Ltd., Tokyo, Japan	1000 mg/ 200 mg	lyophilizer	―
	Facility	Manufacturer	Product		
Gloves	A	O&M Halyard Japan, G.K., Tokyo, Japan	Powder-Free Exam Gloves, PURPLE NITRILE-XTRA		
	B	Medline Japan G.K., Tokyo, Japan	SIGNATURE, LATEX ESSENTIAL		
	C	TAKETORA HOLDINGS Co., Ltd., Yokohama, Japan	Taketora® Nitrile Gloves Long		
	D	O&M Halyard Japan, G.K., Tokyo, Japan	Powder-Free Exam Gloves, PURPLE NITRILE-XTRA		

### Research objects

#### Survey period

The study period was from June 21 to July 16, 2021 before the intervention and from August 16 to September 10, 2021 after the intervention. Both of these periods comprised only the weekday shift, totaling 20 days. To ensure consistency in terms of study conditions, the day shift did not include weekends or holidays to avoid differences in the busyness of the work, and the period was 20 days to minimize bias in terms of the number of patients receiving chemotherapy regimens every two or three weeks.

#### Facilities and subjects in the survey

The survey was conducted at four regional hospitals in the Kanto area (hereafter referred to as A to D). No facility used closed preparation devices for 5-FU and GEM. The characteristics of each facility at the time of the survey are listed in Table 2.

**Table 2:** The characteristics of each facility

Facilities	A	B	C	D
^a^Regional Cancer Hospitals	Yes	Yes	Yes	No
Beds	650	610	400	400
Pharmacists	46	36	20	18
Pharmacists engaged in preparation	28	25	5	13

^a^A medical institution certified by the Ministry of Health, Labour and Welfare in Japan to provide specialized cancer care

The survey participants were pharmacists engaged in preparing anticancer drugs during the period under investigation. The preparers and the collected samples were numbered so that only the authors and a representative of each facility could associate them. In addition, each facility cooperated with us to ensure that the same pharmacist engaged in the preparation, working to the maximum extent during his/her work hours before and after the intervention. When the preparer started the work, a propylene sheet (30 cm × 50 cm) was placed at the center of the safety cabinet, and drugs other than the target drug were prepared as usual. The sheet and all outer gloves used by the preparer during the operation were collected, stored, and delivered to our university at ≤ 10 °C. There were no deviations from the protocol or spillage of large volumes due to an accident.

### Preparation procedures

The preparation procedures recommended in this study ensured that the procedures described in the manual [10] were consistently followed. Based on the results of our previous studies, we recommended implementing procedures that had not been followed previously or using other suggested procedures to reduce the amount of dispersion that had been reported in earlier experiments [8, 12]. We specifically proposed following four steps to reduce the amount of dispersal: Prioritize sequential dissolution and collection when filling multiple vials of lyophilized drugs, perform recap, inject the drug into an infusion bottle with the needle tip pointing upward without filling the needle tip with the drug, and after injecting the drug, remove a small amount of air and place the drug in the syringe before removing the needle. Injection of drug solution into infusion bottle was performed with the needle tip facing upwards without filling the needle tip with the drug solution, removing the needle after injecting the drug solution, injecting a small amount of air, and containing the drug solution in the needle tip within the syringe. When dissolving multiple vials, if the preparer judged that the recommended dissolution method was unsuitable due to problems such as foaming or a substantial volume of solution, the dissolution method could be changed based on the preparer’s choice. To maintain safety, such as to prevent needle sticks during recapping, the guidelines for implementing a safe recapping method in the manual were followed [10]. We provided demonstrations and explanations of the procedures through a video and conducted online training for 14 days for the healthcare workers.

### Analysis by wipe test

After 1 mL of ultrapure water was dripped onto the tested surface, a dry Kimwipe (12 cm × 21.5 cm, Nippon Paper Crecia Co., Ltd., Tokyo, Japan) was cut in half, and each half was used to wipe the surface. The wet Kimwipes were placed in a brown vial. The prepared sheet and each glove were tested independently. ACN (8 mL) was added to each vial to obtain a final volume of 10 mL. The contents of the vial were then mixed by placing it in a shaker for 10 min. The solution was then filtered through a membrane filter (0.20 μm, Millex®-LG 13 mm, Merck Millipore Co., Darmstadt, Germany). The extracted solution (1 mL) was placed in a brown, screw cap vial. IS (10 ng) was added and the concentrations of 5-FU and GEM were determined by LC–MS/MS. The LC–MS/MS conditions and mass spectral parameters are depicted below (Tables 3 and 4, respectively). The limit of quantification for 5-FU and GEM was 1 ng.

**Table 3:** The LC–MS/MS conditions

LC	Equipment: ekspert microLC 200 (eksigent)
	Column: Shim-pack GIST NH2 (75 mm × 1 mm, 3 µm) for 5FU and GEM
	Mobile phase: A: 0.1% (v/v) formic acid in water
	B: Acetonitrile
	Isocratic elution: A 2%, B 98%
	Flow rate: 50 µL/min
	Column temperature: 30℃
	Injection volume: 2 µL
MS	Equipment: QTRAP 5500 (AB Sciex)
	Ion Source: Turbo spray
	Ionization: Electrospray ionization
	Curtain gas: 30 psi
	Collision gas: High
	Ion spray voltage: -4500 V (5FU), 4500 V (GEM)
	Temperature: 300℃
	Measurement mode: Multiple reaction monitoring

**Table 4:** Mass spectral parameters

Analyte	Retention time (min)	Polarity	Precursor ion (m/z)	Production ion (m/z)			
				Quantifier ion (m/Z)	Collision energy (V)	Qualifier ion (m/Z)	Collision energy (V)
5-Fluorouracil	1.63	Negative	128.9	42.0	-26	86.0	-24
Gemcitabine	4.05	Positive	264.1	111.8	27	94.3	63
5-Chlorouracil (IS)	1.59	Negative	144.8	41.9	-30		

The amount of dispersal on gloves was calculated by summing the amount of dispersal on all gloves used by the preparer, using the left and right sides of the gloves as a pair.

### Recovery by the wipe test

To determine the wipe-test recovery, 5-FU and GEM standards were separately dissolved in ultrapure water to make 1 mg/mL solutions. To determine the maximum level of input drug, 10 μL of each solution was placed on a Kimwipe. Subsequently, 10 μL of each drug solution was spread onto the sheets, and the wipe test was performed; five sheets were measured. The wipe test also involved spreading 10 μL of each drug solution onto five nitrile gloves (Powder-Free Exam Gloves, PURPLE NITRILE-XTRA, O&M Halyard Japan, G.K., Tokyo, Japan) and five latex gloves (DIAMOND GRIP PLUS, Ansell Healthcare Japan Co., Ltd., Tokyo, Japan). Each Kimwipe was then placed in a brown vial, 2 mL of ultrapure water and 8 mL of ACN were added, and the mixture was shaken for 10 min. Then, the solution was extracted. IS (10 ng) was added, and the concentrations of 5-FU and GEM were determined by LC–MS/MS. The recovery rate was calculated as the percentage of the maximum level of input drug that was recovered from the sheets and nitrile and latex gloves.

### Statistical analysis

The Wilcoxon signed-rank test was used to determine statistically significant differences (*P* < 0.05). The results were analyzed using the Smirnov–Grubbs rejection test to exclude outliers. Samples below the quantification limit by Fisher’s exact test were used to determine significant differences (*P* < 0.05) before and after the intervention.

## Results

First, the dispersed amount of target anticancer drug during the preparation under study was measured before the intervention. This was estimated again following the procedure designed to reduce the amount of dispersal. We then compared the amount of the target anticancer drug dispersed before and after the recommended intervention.

### Collected samples

A total of 96 sheets and 193 sets of sheets and gloves were collected before the intervention. A total of 83 sheets and 140 sets of sheets and gloves were collected after the intervention. Table 5 shows the number of samples and vials of the collected sheets and gloves in which the target drug was prepared. The number of vials prepared was as follows: 5-FU, 1000 mg/20 mL, 618 before and 409 after the intervention; 5-FU, 250 mg/5 mL, 95 before and 122 after the intervention; GEM, 1000 mg, 167 before and 136 after the intervention; GEM, 200 mg, 285 before and 245 after the intervention. The amount dispersed on the gloves is shown as the sum of the amount dispersed on both gloves (left and right) used by the preparer while preparing the drug.

**Table 5:** Collected samples

		Sheet	Gloves	Vial
5-FU	Before	77	154	713
	After	67	110	531
GEM	Before	72	160	452
	After	61	114	381
Total	Before	96	193	1,439
	After	83	140	1,199

This table was shown the number of sheets and gloves with at least one vial of prepared 5-FU and GEM before and after the intervention. The number of prepared vials was also shown. The number of gloves indicated the number of all gloves collected, including both left and right gloves. Total includes sheets and gloves for which the target drug was not prepared

### Targeted preparers

Forty-one and 39 pharmacists were engaged in preparation work before and after the intervention, respectively. The number of target preparers at each facility and number of target preparers by years of preparation experience are shown in Table 6.

**Table 6:** Targeted preparers

	Total		A		B		C		D	
	Before	After	Before	After	Before	After	Before	After	Before	After
< 5 years	13	13	5	4	3	3	3	2	2	4
5 ≦ years < 10	15	18	10	10	3	7	1	0	1	1
≦ 10 years	13	4	6	1	6	4	2	2	1	1
Total	41	39	21	15	12	14	6	4	4	6

This table was shown the number of target preparers at each facility and the number of target preparers by years of preparation experience

### Recovery by wipe test

The mean ± standard deviation (SD) of the recovery rates was 88% ± 27% for the sheets, 85% ± 4.0% for the nitrile gloves, and 72% ± 24% for the latex gloves in the 5-FU wipe test. In the GEM wipe test, 46% ± 3.0% for the sheets, 44% ± 8.0% for the nitrile gloves, and 71% ± 11% for the latex gloves were recovered.

### Amount of dispersal on the sheets

Data are presented as mean ± SD. The amounts dispersed on the sheets before and after the intervention are shown in Table 7. 5-FU had the highest mean dispersal per prepared vial on the sheets, which then decreased significantly after the intervention (*P* = 0.01). The maximum value and 95% confidence interval of the detected dispersed amount decreased to less than 50%. In addition, no significant differences were found (*P* = 1.00, Fisher’s exact test), but the mean amount of detected 5-FU dispersed per vial on the sheets (excluding those below the quantitation limit) decreased from 440 ± 95 ng before the intervention to 167 ± 42 ng after the intervention, a 62% decrease.

**Table 7:** Amount of dispersal per prepared vial against sheet

	5-FU			GEM		
	Before	After	*P*	Before	After	*P*
*N*	76	67		71	60	
Median (ng)	52	16		2	0	
Mean (mg)	331	127*	0.01 ^a)^	75	34	0.19 ^a)^
SD (ng)	659	271		185	88	
Max (ng)	4267	1867		1078	555	
95% CI (ng)	180–481	61–193		31–118	11–57	
*N* < LOQ	18	16	1.00 ^b)^	33	37	0.11 ^b)^

*N* Number of samples, *SD* Standard Deviation, 95% Confidence Interval (95% CI indicate contamination below the respective limit of quantitation (< LOQ) a) Wilcoxon signed rank test, b) Fisher’s exact test

There was no significant difference in the mean values of dispersed GEM before and after the intervention, but the maximum value and 95% confidence interval of the detected dispersed amount decreased similar to that of 5-FU. The percentage of sheets below the quantitation limit after the intervention was 62%, which was increased from the value before intervention (46%). In addition, the mean amount of GEM dispersed per vial on the sheets (excluding those below the quantitation limit) decreased from 140 ± 235 ng before the intervention to 85 ± 123 ng after the intervention, a 61% decrease. While no significant differences were found (*P* = 0.11, Fisher’s exact test), the number of sheets below the quantitation limit increased and the detected amount of dispersal showed a decreasing trend after the intervention.

**Fig. 1:**
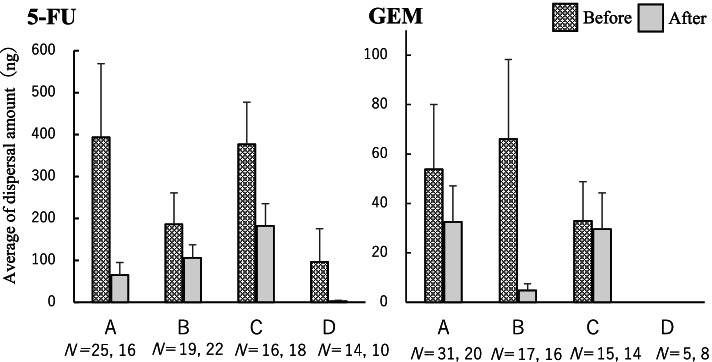
Average amount of dispersal on sheets per facility (ng). Mean and standard error of 5-FU and GEM dispersal on sheets per prepared vial at each facility (**A–D**) are shown. The number N indicates the number of samples collected at each facility before and after the intervention

Figure 1 shows the amount of dispersal per prepared vial at each facility. The SD was large, and there was no significant difference between the mean values of the dispersal amounts before and after the intervention. Moreover, no facility reported an increase in mean dispersal amount after the intervention. There was no significant difference among the facilities, and the dispersal per prepared vial was higher for 5-FU than for GEM at all facilities.

**Fig. 2:**
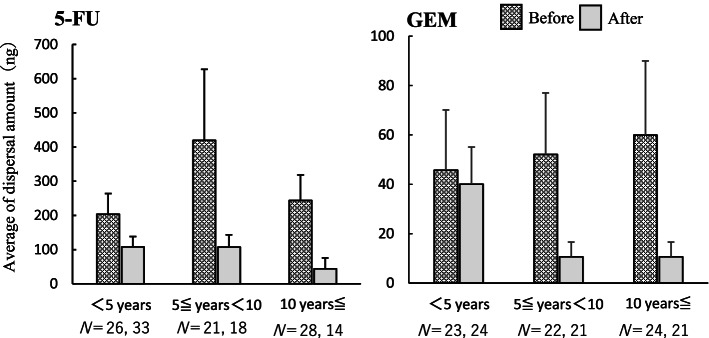
Average amount of dispersal on sheets for each year of experience (ng). Mean and standard error of 5-FU and GEM dispersal on sheets per prepared vial for each year of experience (< 5 years, 5 ≤ years < 10, ≥ 10 years) are shown. The number N indicates the number of samples collected for each year before and after the intervention

The mean amount of dispersal per prepared vial according to the years of preparation experience of the pharmacists is shown in Fig. 2. For novice pharmacists, after the intervention, no increase in the dispersed amount of 5-FU was observed, but the SD of the dispersed amount tended to decrease. Furthermore, dispersal tendency did not decrease with years of experience. Instead, this tendency increased in pharmacists with more than five years of preparation experience.

### Amount of dispersal on the gloves

Table 8 shows the dispersal amounts on the gloves before and after the intervention. 5-FU was the drug with the highest mean amount of dispersal on the gloves per prepared vial. There was no significant difference between before and after the intervention for any of the drugs and the amount of dispersal on the gloves. However, the maximum amount of dispersal for 5-FU decreased to less than 50% after the intervention. The proportion of gloves below the quantitation limit increased more than 1.5-fold for both drugs after the intervention. In particular, with GEM, gloves below the limit of quantitation were significantly increased after the intervention (*P* = 0.03, Fisher’s exact test).

**Table 8:** Amount of dispersal per prepared vial for gloves

	5-FU			GEM		
	Before	After	*P*	Before	After	*P*
*N*	76	67		71	60	
Median (ng)	180	48		11	8	
Mean (ng)	510	326	0.05 ^a)^	138	171	0.66 ^a)^
SD (ng)	847	574		308	403	
Max (ng)	4983	2931		1699	1754	
95% Cl (ng)	316–704	184–468		66–211	67–276	
*N* < LOQ	10	15	0.18 ^b)^	10	18*	0.03 ^b)^

*N* Number of samples, *SD* Standard Deviation, 95% Confidence Interval (95% CI)indicate contamination below the respective limit of quantitation (< LOQ) a) Wilcoxon signed rank test, b) Fisher’s exact test

**Fig. 3:**
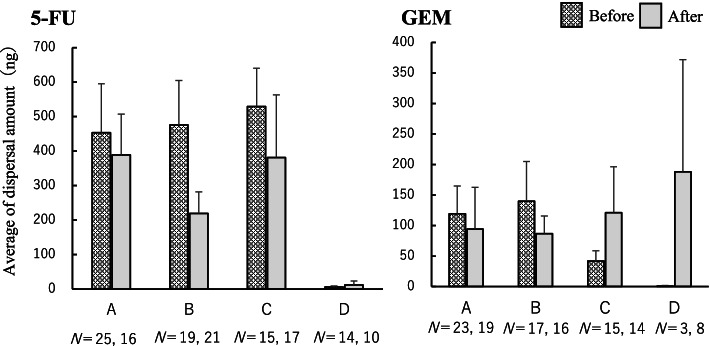
Average amount of dispersal on gloves per facility (ng). Mean and standard error of 5-FU and GEM dispersal on gloves per prepared vial at each facility (**A–D**) are shown. The number N indicates the number of samples collected at each facility before and after the intervention

The amount of dispersal per prepared vial for each facility is shown in Fig. 3. The SD was large, and there were no significant differences in the mean values of the dispersal amounts before and after the intervention. There was no significant difference among the facilities, and the dispersal amount per prepared vial was higher for 5-FU than for GEM in all facilities.

**Fig. 4:**
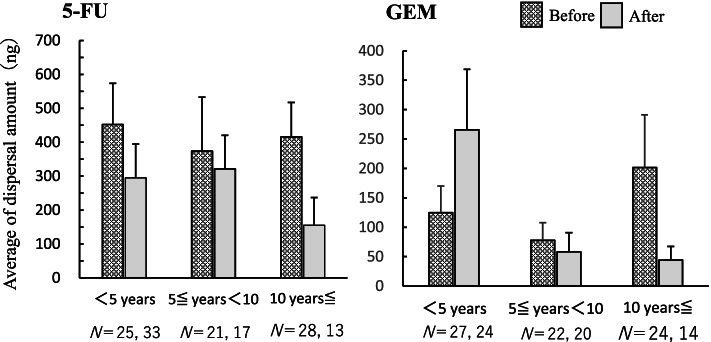
Average amount of dispersal on gloves for each year of experience (ng). Mean and standard error of 5-FU and GEM dispersal on gloves per prepared vial for each year of experience (< 5 years, 5 ≤ years < 10, ≥ 10 years) are shown. The number N indicates the number of samples collected for each year before and after the intervention

The mean values of the dispersal volume per prepared vial by years of preparation experience are shown in Fig. 4. There was no significant difference between the mean values of the dispersed amount before and after intervention.

## Discussion

The number of 5-FU preparations was more than 10 times higher than that of GEM in terms of dispersal per prepared vial detected on sheets and gloves. The amount of dispersal on the sheets was significantly reduced by recommending appropriate preparation techniques. Inclusion of infuser pump preparations for 5-FU may also have affected the higher dispersal rate. When preparing the infuser pump, it is expected that the number of times that air is removed and the scale is adjusted before injecting the drug in the open system is high, and the recapping recommended in this study may have influenced the reduction in the amount of dispersal. The manual [10] does not necessarily state that recapping should be performed, and the facilities included in this study did not have a particularly strong recommendation. Although it is possible that some preparers had been performing recapping before this survey, we recommended recapping again for all operations when preparing the target drugs, which may have contributed to the reduction in the amount of dispersal. There were no incidents of needle sticks. In our previous experiments, it was suggested that air venting or scale adjustment without recapping, accompanied by plunger depression, can cause a large amount of dispersal from the needle tip [12]. However, the amount of dispersal on the gloves was not significantly reduced, and it is necessary to recognize that a certain amount of dispersal is observed even when appropriate preparation procedures are utilized. We considered both gloves to be close to the source of the spill for both 5-FU and GEM because the source of leakage, where the solution becomes an open system, is likely to be the rubber stopper of the vial after the needle is inserted and the needle tip after the drug solution is collected. In other words, the gloves were contaminated first, and the solution that did not get on the gloves contaminated the sheet. The large amount of dispersed solution on the sheet suggests that widespread contamination (e.g., splashing) is occurring, although it cannot be visually observed. Conversely, the decrease in contamination on the sheet can be attributed to a decrease in more extensive contamination. The lack of reduction in glove contamination can be attributed to the fact that proper implementation of the preparation technique reduced widespread contamination but did not lead to a significant reduction in glove contamination, which is closer to the source of the contamination.

With the results for GEM, the amount of dispersal on sheets and gloves was not significantly reduced by the recommended appropriate preparation techniques. The lack of difference in the results for sheet contamination for GEM and the amount of glove contamination for either agent may not have reached statistical significance due to the magnitude of the error. However, the percentage of sheets that were below the quantitation limit increased after the intervention, suggesting that the intervention contributed to a reduction in the amount of dispersal. The reason for the lack of significant differences may be that there were many occasions when the recommended dissolution method could not be used and that the number of times that recapping was performed or the appropriate drug-injection technique was used, which greatly affected the overall amount of dispersal in our experimental validation [12], was small. There was no difference in the amount of dispersal on gloves before and after the intervention, and it should be recognized that dispersal was observed even with appropriate preparation techniques.

In facilities other than C, the GEM was lyophilized, and it was expected that the recommended drug dissolution method, recapping, drug injection method, and manipulation at the time of needle removal would affect the results. In facility C, a liquid vial formulation of GEM was used, but the effect on the amount of dispersal per vial during preparation was the same as that of the lyophilized formulation. We hypothesized that the amount of GEM dispersal at facility C, which used liquid formulations, was lower than at the other facilities. However, there was no clear difference in the amount of GEM dispersal between the lyophilized and liquid formulations, although this was a comparison among the other facilities. The reason for the lack of reduction in GEM dispersal at facility C could be that there were fewer procedures that could be improved through education, since the procedures were simpler due to the liquid formulation.

Regarding the differences in the amount of dispersal among the facilities, from the overview of each facility, it was recognized that there were differences in the facility size, number of pharmacists engaged in the preparation, and drugs employed, but there were no obvious differences in the amount of dispersal of the target anticancer drugs in this study. The SD of the dispersal amount was also large, suggesting that the amount of dispersal during the preparation of anticancer drugs is more influenced by individual factors of the preparer than by the preparation environment.

A comparison of the amount of dispersed drug per prepared vial according to the number of years of preparation experience showed that there was no correlation between the number of years of experience and dispersed amount of the target anticancer drug. However, the amount of dispersed drug per sheet tended to be higher in pharmacists with more than five years of experience. Our past findings [8] have shown that there is a lack of ongoing education on preparation. Therefore, we believe that pharmacists with more years of experience may deviate from proper preparation procedures. We contend that review of proper preparation procedures has resulted in lower dispersal rates.

Yoshida et al. conducted a wipe survey of anticancer drugs (cyclophosphamide, 5-FU, platinum, and GEM) using 0.03 mol/L sodium hydroxide solution and tissues [11]. Shionogi Pharma Inc. developed a sampling sheet method, which has become a mainstream method for environmental monitoring. However, the recovery rates of both methods have not yet been publicized. In the report on their wipe analysis method, Takano et al. indicated recoveries of 45–82% for GEM and significantly lower than 20% for 5-FU [13]. The method shown by Takano et al. uses methanol as the wipe-off solvent, which we believe results in a lower recovery rate for 5-FU than for GEM. In the present study, because water was used as the wipe-off solvent, the recovery of GEM was probably lower than that of 5-FU because GEM (log*P*_ow_ = 0.14) is more hydrophobic than 5-FU (log*P*_ow_ = -1.00) and is retained on the sheets and gloves.

We could estimate the level of contamination using a simple wipe test. The wipe test of gloves was also established, and it was confirmed that quantitative accuracy was maintained even when the recovery depended on the composition of the gloves. Although the quantitative accuracy is not high for SDs greater than 10%, it is possible to estimate the amount of the target anticancer drug remaining on the sheets and gloves. The wipe test used in this study is expected to be used for future environmental monitoring.

In this study, we quantitatively evaluated the amount of dispersal in the BSC and on gloves while preparing the anticancer drugs 5-FU and GEM, for which a CSTD is not sufficiently widespread despite the frequent use of these drugs. We also showed that the amount of dispersal could be reduced by implementing appropriate preparation techniques that immediately impact individual awareness. It was also shown that the amount of dispersal was higher with more experienced preparers. Although teaching is provided to newcomers for preparing anticancer drugs, only 23% of facilities provide continuous training [8], suggesting that it would be helpful to periodically provide continuous training on appropriate preparation techniques in the future. For gloves, even with appropriate preparation procedures, it is necessary to be mindful that a certain amount of anticancer drug will be dispersed, although the contamination may not be clearly visible. Therefore, it is advisable to change gloves as often as possible.

A key limitation of this study is that the preparers and regimens before and after the intervention were not necessarily the same. In addition, a control group was not established, and it is not always possible to show that there is a strong causal relationship between the implementation of appropriate preparation procedures and the amount of dispersal. To address these limitations, we calculated the amount of dispersal per vial using the same preparers as much as possible and recorded the number of vials of the target drug prepared by each individual. In addition, a video demonstration of the preparation technique was used to ensure that the intervention was properly implemented. However, the number of vials used per patient was higher for 5-FU than for GEM. Furthermore, this study did not collect data on the rate of infuser pump filling for the 5-FU preparation and the number of units of each drug prepared per patient.

The results of this study indicate the importance of appropriate preparation techniques. However, it is difficult to maintain the amount of dispersal below the quantitation limit and such techniques are not reliable exposure control measures. Hence, it is important to implement them in combination with other exposure control measures. In addition, there are drawbacks to tissue management controls and these shortcomings provide evidence for the need to use instruments such as CSTDs to further reduce the amount of dispersal.

## Conclusions

Quantitative amounts of anticancer drugs were shown to be dispersed in the preparation of 5-FU and GEM in our study. The difference in the amount of contamination before and after the intervention was significantly reduced only for the contamination of sheets with 5-FU. There was no decrease in the amount of contamination of gloves. There were also no differences among medical facilities. Even if the preparation procedure is properly implemented, not all dispersed amounts can be kept below the quantitation limit, indicating the need for a combination of education and engineering controls, such as the use of sealed connection devices.

## Data Availability

All data supporting the conclusions are included in the article.

## Abbreviations

5-FU: 5-Fluorouracil
GEM: Gemcitabine
5-CU: 5-Chlorouracil
IS: Internal standard
SD: Standard deviation
NIOSH: National Institute for Occupational Safety and Health
ASHP: American Society of Health-System Pharmacists
CSTD: Closed system drug-transfer device
BSC: Biological safety cabinet
PPE: Personal protective equipment
